# Working memory updating for free items and for item-to-context bindings: When attention is enough and when gating is needed

**DOI:** 10.3758/s13421-025-01728-7

**Published:** 2025-05-07

**Authors:** Yoav Kessler, Sam Verschooren

**Affiliations:** 1 Department of Psychology, Ben-Gurion University of the Negev, P.O.B. 653, 84105 Beer-Sheva, Israel; 2 Ghent University, Ghent, Belgium; 3 Duke University, Durham, NC USA

**Keywords:** Working memory, Updating, Gating

## Abstract

A well-supported working memory (WM) model holds that a “gate” separates the content of WM from information that does not need to be maintained or manipulated. Previous research suggests that switching between opening and closing this gate incurs a response-time cost, reflecting controlled cognitive effort. However, the exact nature of this cost remains debated. Some studies find that closing the gate is more costly than opening it, while in other studies these costs are comparable. Using an intertrial interval manipulation in the reference-back paradigm, we show that the larger cost of gate-closing is not an intrinsic feature of WM control, but is instead influenced by the automatic retention and removal of stimulus- and response-related information in WM. This finding indicates that WM is automatically but transiently updated with information for which attention-consuming processes such as response selection take place, challenging the prevailing view that WM updating is always effortful and controlled. Crucially, our findings reveal that updating individual items occurs rapidly and automatically when a single item is maintained. In contrast, updating bindings between items and their context is a slower, effortful process that requires gating. These results reconcile conflicting views regarding the nature of working memory encoding and updating.

## Introduction

Working memory enables us to maintain information over short-term durations, typically around a few seconds, and to use this information in the service of our ongoing thought, reasoning, and behavior. The maintained information may stem from the outside world, being the product of perception, or from the internal environment, being thoughts or items that are brought to mind by retrieval from long-term memory. Each of these external and internal sources may feed working memory with goal-relevant information, but also may include distractions irrelevant to the task at hand. To efficiently use the severe capacity limitations of working memory, being around three to four items only (Cowan, 2001), relevant information should be maintained, whereas irrelevant information must be filtered out.

A fundamental question, therefore, is which items get into working memory and how the system distinguishes relevant from irrelevant information. At this stage, by “getting into working memory,” we refer to both encoding new information from scratch and updating existing representations by substituting old information with an updated one (Ecker et al., 2010). Two views have been suggested. On the one hand, gating models regard updating as selective, controlled, and resource-consuming (Braver & Cohen, 2000; Frank et al., 2001). On the other hand, the notion of working memory as closely related to attention implies that encoding is an automatic by-product of attending to information (Cowan, 1988; Kiyonaga & Egner, 2014; for review see Oberauer, 2019). The present work will demonstrate that the two types of updating coexist and map this distinction to the difference between updating items and updating item-to-context bindings. We will discuss each of these views shortly.

### Gating models

Gating models (Braver & Cohen, 2000; Frank et al., 2001) assume that maintenance is selective and that encoding is controlled by a “gate” that regulates the flow of information into working memory. This gate balances between the need to keep working memory up to date with relevant information and the need to shield the maintained information against the potential interference by task-irrelevant input. To accommodate these opposing demands, computational models suggest that input selection into working memory and shielding its contents are achieved by a selective gating mechanism. Specifically, the content of working memory is separated from perceptual and long-term memory representations by a dynamic gate that controls the flow of information into working memory. Opening the gate enables relevant information to enter working memory and update its content, whereas closing it results in filtering out irrelevant input while protecting the existing contents of working memory from interference. Input selection into working memory is therefore achieved by controlling the state of this gate.

According to the prefrontal cortex basal-ganglia working memory model, an influential gating model, the prefrontal cortex is crucial for the robust maintenance of working memory representations.^1^ The selective gating mechanism is neurally implemented in this model by multiple parallel loops that connect the prefrontal cortex and the basal ganglia (Frank et al., 2001). The gate is closed by default, enabling robust maintenance of information by filtering out potential distractors. This is achieved by tonic firing of the substantia nigra, which inhibits the excitatory connections between the prefrontal cortex and the thalamus. Firing of “Go” neurons in the dorsal striatum disinhibits the prefrontal-thalamic connection and hence leads to transient “opening” of the gate to working memory, allowing for perceptual information to flow from perception to working memory.

### The reference-back paradigm

**Fig. 1:**
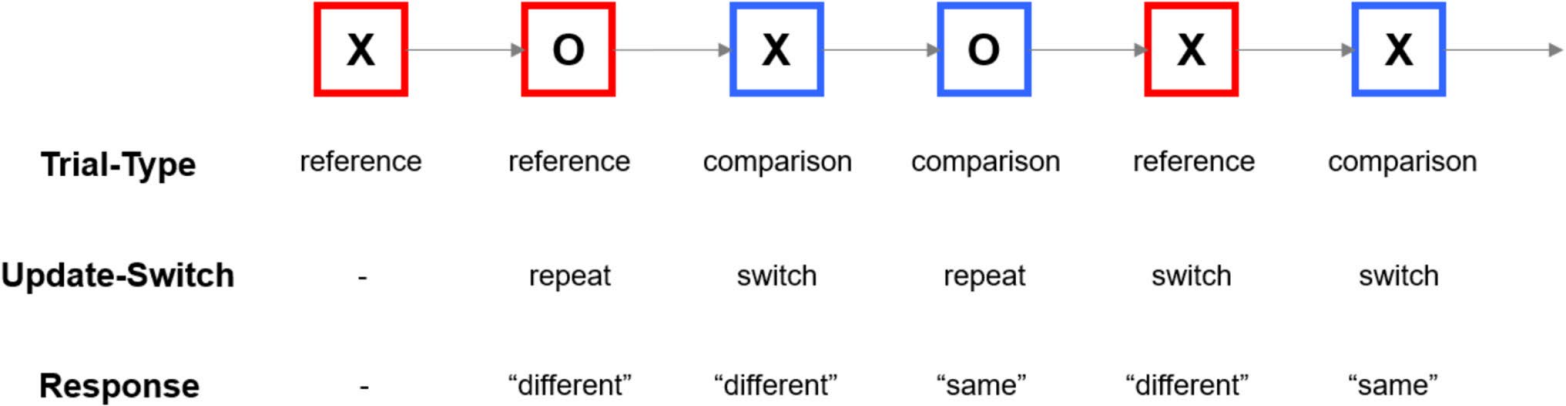
The reference-back paradigm. Red and blue squares represent reference and comparison trials, respectively. The letter on screen needs to be compared with the one that appeared in the most recent red frame, yielding a “same” or “different” response. Update-switch refers to whether the trial-type repeated or alternated compared with the previous trial. Note that update-switch and response are only meaningful starting from the second trial in the sequence. (Color figure online)

The gating model implies that updating is carried out by a cascade of (potentially time-consuming) processes that include opening the gate to working memory, encoding the new information, and re-closing the gate. The *reference-back paradigm*, which is also utilized in the present study, has been developed to tease these sub-processes apart (Nir-Cohen et al., 2020; Rac-Lubashevsky & Frank, 2021; Rac-Lubashevsky & Kessler, 2016a, 2016b; Verschooren et al., 2021; for review see Trutti et al., 2021). On each trial of this task, either the letter “X” or “O” is presented (see Fig. 1), surrounded by a red or blue frame. Participants are required to indicate whether the letter is the same as or different from the one that appeared in the previous red frame. To perform correctly, they need to maintain in working memory the letter that appears in the most recent red frame, and update this letter whenever a new red frame is presented. Trials involving red frames are termed “reference trials,” since the stimulus (letter) presented in them will serve as a reference to which future stimuli will be compared. Trials involving blue frames are termed “comparison trials,” in which participants merely need to compare the previous reference stimulus to the one currently on screen. Consequently, both trial-types require a same/different decision. However, reference trials also require to update working memory with the letter that appear in them, because this letter would serve as a reference to which the following trials would be compared. Conversely, stimuli that appear in comparison trials (namely, in blue frames) should not be updated into working memory.

With the gating model in mind, this paradigm was designed to enable researchers to examine the behavioral costs in response time and error rate of the sub-processes involved in working memory updating, as well as their neural correlates. As explained above, both reference and comparison trials require a same/different decision, but reference trials also demand updating. Accordingly, the difference in performance between the two conditions corresponds to the process of updating—namely, replacing one item with another. Also, reference trials (in which updating takes place) require the gate to working memory to be open, whereas comparison trials (in which updating should be prevented) require a closed gate to prevent the stimulus that appears inside them from entering working memory. Therefore, switching from a reference trial to a comparison trial involves gate-closing, whereas switching in the other direction (i.e., from a comparison to a reference trial) requires gate-opening. The gate-closing contrast is calculated by subtracting performance or brain activation in trials in comparison trials that were preceded by comparison trials (i.e., maintaining a closed gate) from comparison trials that were preceded by reference trials and hence needed to close the gate. Similarly, the gate opening contrast is calculated by subtracting trials on which participants repeat a reference trial (i.e., maintaining an open gate) from trials on which they switch to a reference trial (i.e., gate opening). While past research has shown that switching the “state” of the gate to working memory is associated with a marked response-time cost (Kessler & Oberauer, 2014, 2015), the reference-back paradigm enables to differentiate between gate-opening and gate-closing in an orthogonal manner and examine their properties independently. Importantly, typical findings in this task demonstrate robust behavioral costs associated with updating, gate-opening, and gate-closing, supporting the notion that updating involves a controlled, time-consuming, and effortful sequence of processes.

### Automatic encoding/updating

Gating models posit that the default state of working memory is “maintenance”—namely, shielding its content against potential interference from internal or external information. The idea that updating is a transient process that overcomes this shielding fits well with the individual differences literature, which regards updating as a controlled, effortful, and goal-directed process (Friedman & Robbins, 2022; Miyake et al., 2000). However, this approach seems to contrast with another common view in the literature, which regards working memory as being closely related to selective attention (Cowan, 1988; Kiyonaga & Egner, 2014; for review, see Oberauer, 2019). According to this view, attending to an item may lead to entering it into working memory without needing an additional process of encoding. While there are some cases where attention is insufficient (Fu et al., 2023), this view implies that the contents of working memory rapidly change whenever we shift our attention from one item to another. This contrasts with the idea that the gate to working memory is closed by default and that updating and gate-opening are time-consuming processes necessary for maintaining information in working memory.

In line with this view, a recent study by Kessler and colleagues (2023) showed that in some cases, updating working memory with new information is indeed *faster* than not updating it, suggesting that updating may be a by-product of attention. A new version of the reference-back task was used. The set size—namely, the number of items that had to be maintained in WM throughout the task—was manipulated to be either 1 or 2. When the set size was a single item, in each trial a letter (X or O) appeared within a red or blue frame. Unlike the standard reference-back task, the participants were not required to make a same/different response in each trial. Instead, they were asked to indicate the identity of the presented letter by pressing one key if X was presented and another if O was presented. As in the standard reference-back task, after a varied number of trials, the participants had to recall the letter that appeared within the most recent red frame. The reasoning was that to report this letter correctly, participants needed to update their working memory when a red frame was presented and not to update when a blue frame was presented. Unlike the “standard” reference-back task, in the set size = 1 condition, participants were *faster* to respond to letters within red frames (“update trials”) than to letters within blue frames (“no-update trials”). The authors interpreted this finding as evidence that updating may automatically take place as part of response selection. Specifically, deciding whether the stimulus is X or O requires attending to the stimulus, and this act of attention is sufficient to bring the information into working memory without an additional encoding/updating process. Since updating is an obligatory outcome of response selection, letters appearing in blue frames (and should not be memorized further) must be actively removed from working memory. This added removal process (Lewis-Peacock et al., 2018) leads to slower responses in the no-update condition.

A reversed pattern emerged in the set size = 2 condition of Kessler and colleagues’ (2023) study. In this condition, two frames appeared on the screen throughout the sequence of trials, one on the left and the other on the right side of the screen. In each trial, one letter was presented in one of these frames, and the participants needed to indicate whether it is X or O using a key press. After a random number of trials, participants had to report two items—the letters that appeared in the most recent red frame on each side of the screen. Accordingly, to perform this task correctly, participants had to constantly maintain two working memory items corresponding to the two frames. In this condition, update trials (red frames) performance was *slower* than in no-update trials (blue frames). Note that in both set sizes only one item was presented in each trial. However, the total number of items maintained, being one or two, affected the direction of the update effect.

To reconcile these findings, it was suggested that whenever one items is maintained in working memory, its updating is obligatory and takes place as part to attending to information. However, keeping more than one item in mind requires the maintenance of bindings between items and their context. In this case, the binding is between a letter and its corresponding frame (e.g., whether X corresponds to the right frame and O to the left, or the other way around). In this situation, updating entails modifying not only the item but also its binding to the context. The updating cost observed in this condition implies that unlike updating an “unbound item” (i.e., in set size = 1), the updating of item-to-context bindings is slow and effortful.

The findings discussed above call into question the interpretation of the typical finding in the “standard” reference-back task (Rac-Lubashevsky & Kessler, 2016a, 2016b), in which only one item needs to be maintained (namely, the letter in the most recent red frame), but updating is slower than not-updating. The results of the present study will offer a new interpretation for this finding by reconciling effortful updating, where gating is required, and automatic updating, where gating is unnecessary. We will discuss this interpretation in detail in the General Discussion.

### The present study

So far, we have examined the effect of updating vs. not updating information into working memory. In addition to the updating effect, previous research with the reference-back paradigm has documented evidence for response-time and error-rate costs associated with alternating between the update and no-update conditions. These costs were interpreted as reflecting gate-opening and gate-closing (Rac-Lubashevsky & Kessler, 2016a, 2016b; Verschooren et al., 2021; for neural correlates, see Nir-Cohen et al., 2020, 2023; Rac-Lubashevsky & Frank, 2021; Rac-Lubashevsky & Kessler, 2018; Rempel et al., 2021; Yu et al., 2024; see Trutti et al., 2021, for review). To avoid theoretical confusion, we decided to use the theoretically-neutral term “update-switch” to refer to the effect of alternating between updating and not-updating, rather than gate-opening and gate-closing.

In principle, the duration of update-switch costs should reflect the ease or difficulty of the gate-opening and gate-closing operations, and hence is theoretically meaningful. Interestingly, in some studies, the cost of switching from update to no update (“gate-closing”) and from no-update to update (“gate-opening”) costs were comparable. In other studies, however, the cost of switching to no-update (“gate-closing”) was larger. Both a larger gate-closing cost, which implies that it is harder to close the gate than to open it, and comparable costs, seem to be at odds with the gating model described above. This is because this model explicitly assumes that the closed-gate state to be the default one (Frank et al., 2001). Under this assumption, it is expected that returning to this default state should be easier than moving to the state that requires separate activation. However, the empirical inconsistencies need first to be systematically examined and clarified. This was the starting point for the present study.

One difference between studies that found comparable vs. asymmetrical update-switch costs was the length of the intertrial interval. Specifically, studies that used relatively short intertrial intervals of around 1 s showed a larger gate-closing cost (Rac-Lubashevsky & Kessler, 2016a, 2016b; Verschooren et al., 2021), whereas studies that use longer intertrial intervals, around 4 s or more, showed comparable opening and closing costs (Nir-Cohen et al., 2020; Rac-Lubashevsky et al., 2017). See Table 1 for details.

**Table 1:** Gate-opening and gate-closing costs in previous studies, arranged by ITI

	ITI (ms)	Gate-opening cost (ms)	Gate-closing cost (ms)	Difference (ms)
Verschooren et al. (2021)	**350**	**68**	**125**	**57**
Rac-Lubashevsky and Kessler (2016b)**, Exp. 3**	**1,050**	**106**	**189**	**83**
Rac-Lubashevsky and Kessler (2016a)	**1,800**	**93**	**143**	**50**
Rac-Lubashevsky et al. (2017), Exp. 1	2,000	152	139	− 13
Rac-Lubashevsky et al. (2017), Exp. 2	4,500—RT^a^	89	85	− 4
Nir-Cohen et al. (2020)	2,000 to 8,000^b^	72	52	− 20

Bolded lines indicate that the difference between the costs was statistically significant. ^a^ In this experiment, the ITI was 4,500 ms minus the RT of that trial. RTs averaged 750 ms approximately. ^b^ In this imaging experiment the ITI randomly varied between trials and was set to 2, 4, 6, or 8 s

At first glance, this difference in the intertrial interval may look negligible and not directly related to the questions at hand. However, the present study revealed that this seemingly nuisance detail has important empirical and theoretical implications. Specifically, we show that the interaction between the intertrial interval and update-switching is reliable and that this interaction is driven by a specific sequence of conditions that involve the removal of irrelevant information from working memory. In light of these findings, we offer a new interpretation of previous results with the reference-back task, which has important implications for the debate over the automatic vs. controlled nature of working memory updating discussed above.

## Experiment 1

The goal of Experiment 1 was to replicate the observation regarding the effect of the intertrial interval on the interaction between updating and update-switching within a single experiment and to establish a robust empirical picture. To this end, we systematically manipulated the intertrial interval to be either 1 s (short) or 4 s (long), based on its duration in previous studies.

### Method

#### Participants

Twenty undergraduate (16 women; age range: 19–26 years, mean = 22.95 years, *SD* = 1.67) students from the Ben-Gurion University of the Negev participated in the study in exchange for course credit. Data collection for this experiment was completed in March 2018. No formal power analysis was conducted. This sample size was based on our previous observations of the gating-cost asymmetry (Rac-Lubashesky & Kessler, 2016a, 2016b; Verschooren et al., 2021), and the observation that the update-switch asymmetry is found in about 75% of the participants (66/88; Rac-Lubashesky & Kessler, 2016b). All the participants were right-handed and reported having no neurological problems or learning disabilities. The study was approved by the Department of Psychology Ethics Committee, Ben-Gurion University of the Negev. The current findings apply most directly to this subpopulation, but we assume that it does not differ in the relevant characteristics from the general population and that our findings generalize.

#### Procedure and stimuli

The experiment was presented with E-Prime software (Psychology Software Tools, Pittsburgh, PA). The experimental session started with a 40-trial practice block. On the first trial of each block, one of two stimuli (i.e., the letter “X” or “O”) appeared on screen in a red frame and participants were instructed to keep this stimulus in mind (i.e., update working memory with this stimulus). On each subsequent trial, either the letter “X” or “O” appeared within a red or blue frame, which indicated whether the current trial was a reference or comparison trial. On comparison trials, participants compared the stimulus that was presented on the screen with the one that was currently in working memory and responded with a “same” of “different” response. On reference trials, participants did the same, but in addition replaced the stimulus currently in working memory with the one on screen (until the next reference trial). Each stimulus was presented for 2,000 ms or until response. In the practice block, a constant intertrial interval of 2,500 ms was used.

After the practice block, we administered 16 experimental blocks. In half of these blocks the intertrial interval was 1,000 ms and in the other half it was 4,000 ms. The order of these blocks was random. Apart from this difference in intertrial interval, the trials were identical to those in the practice block. The first stimulus (“X”/“O”) in each block always appeared within a red frame (reference trial) and was presented for 2,000 ms. Thirty-five more trials followed, in which both trial-types (reference/comparison) and responses (same/different) appeared randomly with equal probabilities. Each stimulus appeared until a response was indicated. Participants responded with the “p” key for “same” and the “q” key for “different” responses.

The experiment had a 2 (trial-type: comparison, reference) × 2 (update-switch: repeat, switch) × 2 (intertrial interval: short, long) within-subject design. We presented participants with 648 trials in total, 81 trials per cell of the design. Each session lasted for around 45 min.

#### Data analysis

Data preprocessing, visualization, and analysis was carried out in R Studio (RStudio Team, 2020; Version 1.1.456), using the *tidyvers*e (Wickham et al., 2019), *emmeans* (Lenth, 2019), *effectsize* (Ben-Shachar et al., 2020), *datawizard* (Patil et al., 2022), *afex* (Singman et al., 2024), *parameters* (Lüdecke et al., 2020), *bayestestR* (Makowski et al., 2019), and *tidylog* (Elbers, 2024) packages. All data and analysis scripts can be found on our Open Science Framework profiles (https://osf.io/7zspj/). The practice block, as well the first trial of each block, were removed from the analysis. Response-time outliers were removed in two steps. First, we filtered out all trials with response-times faster than 100 ms or slower than 10,000 ms. Then, trials that deviated more than 3 standard deviations from the mean of their condition, within each participant were removed. For the response-time analysis, error trials and trials preceded by an error were removed. A repeated-measures analysis of variance (ANOVA) was conducted on the aggregated data. Violation of sphericity for the four-way model was adjusted using the Greenhouse–Geisser correction. Bayes factors (BFs) were calculated using a *p*-value to BF conversion, which is an approximation to BIC-to-BF conversion, using the rules suggested by Wagenmakers (2022).

#### Transparency and openness

All data and analysis scripts are available on Open Science Framework (https://osf.io/7zspj/). This study’s design and its analysis were not preregistered.

### Results

#### Response times

**Fig. 2:**
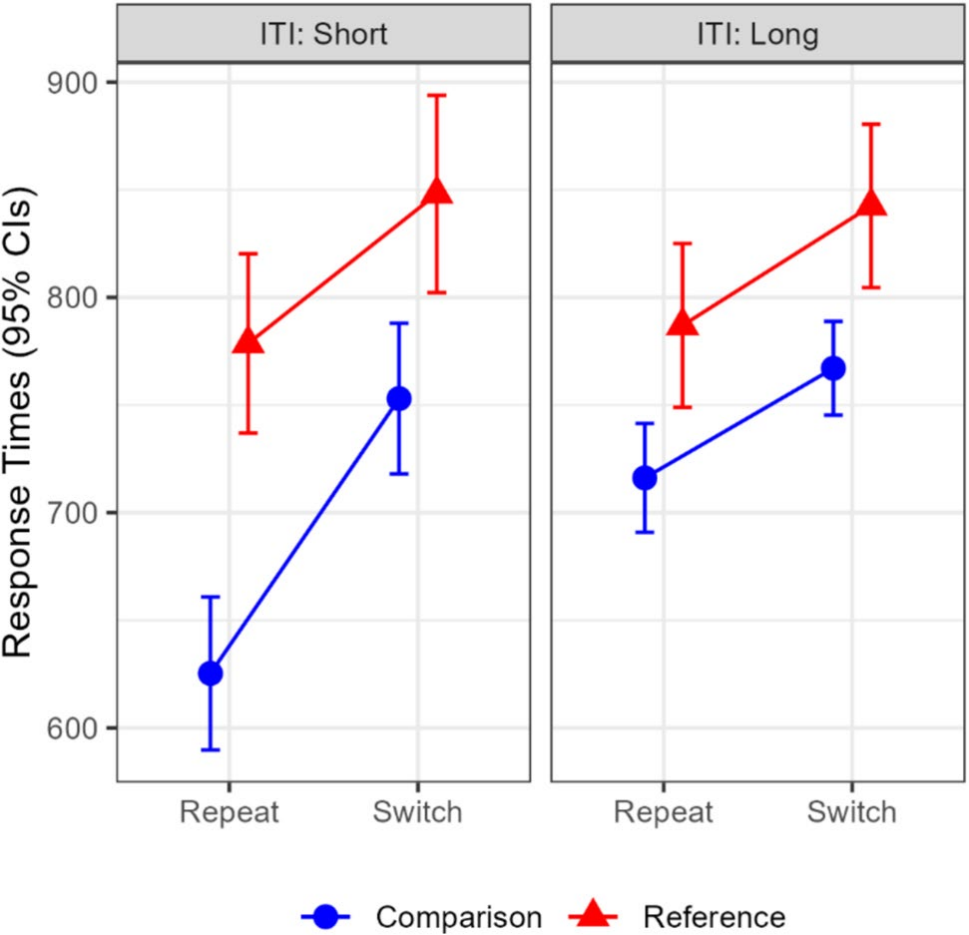
Experiment 1: Mean response times by update-switch and intertrial interval. CI = confidence interval; ITI = intertrial interval. Error bars denote 95% within-subject confidence intervals

A repeated-measures ANOVA was conducted with intertrial interval, trial-type, and update-switch. Significant main effects were found for trial-type, *F*(1,19) = 60.92,* MSe* = 6391.60,* p* < 0.001, η_p_^2^ = 0.76, BF = 3.09 × 10^5^, and for update-switch, *F*(1,19) = 39.36,* MSe* = 5853.99,* p* < 0.001, η_p_^2^ = 0.67, BF = 1.48 × 10^4^. Significant two-way interactions were observed between intertrial interval and trial-type, *F*(1,19) = 6.96,* MSe* = 3746.79,* p* = 0.016, η_p_^2^ = 0.27, BF = 4.60, and between intertrial interval and update-switch, *F*(1,19) = 8.63,* MSe* = 2382.66,* p* = 0.008, η_p_^2^ = 0.31, BF = 8.81. Lastly, the three-way interaction was significant, *F*(1,19) = 5.35,* MSe* = 1841.90,* p* = 0.032, η_p_^2^ = 0.22, BF = 2.32 (see Fig. 2). To probe this interaction, we examined the simple two-way interactions between update-switch and trial-type within each intertrial interval separately. This interaction was close to significance in the short interval, *F*(1,19) = 3.82,* p* = 0.066, η_p_^2^ = 0.17, BF = 1.14, but absent in the long interval, *F*(1,19) = 0.13,* p* = 0.724, η_p_^2^ < 0.001, BF = 0.24.

**Fig. 3:**
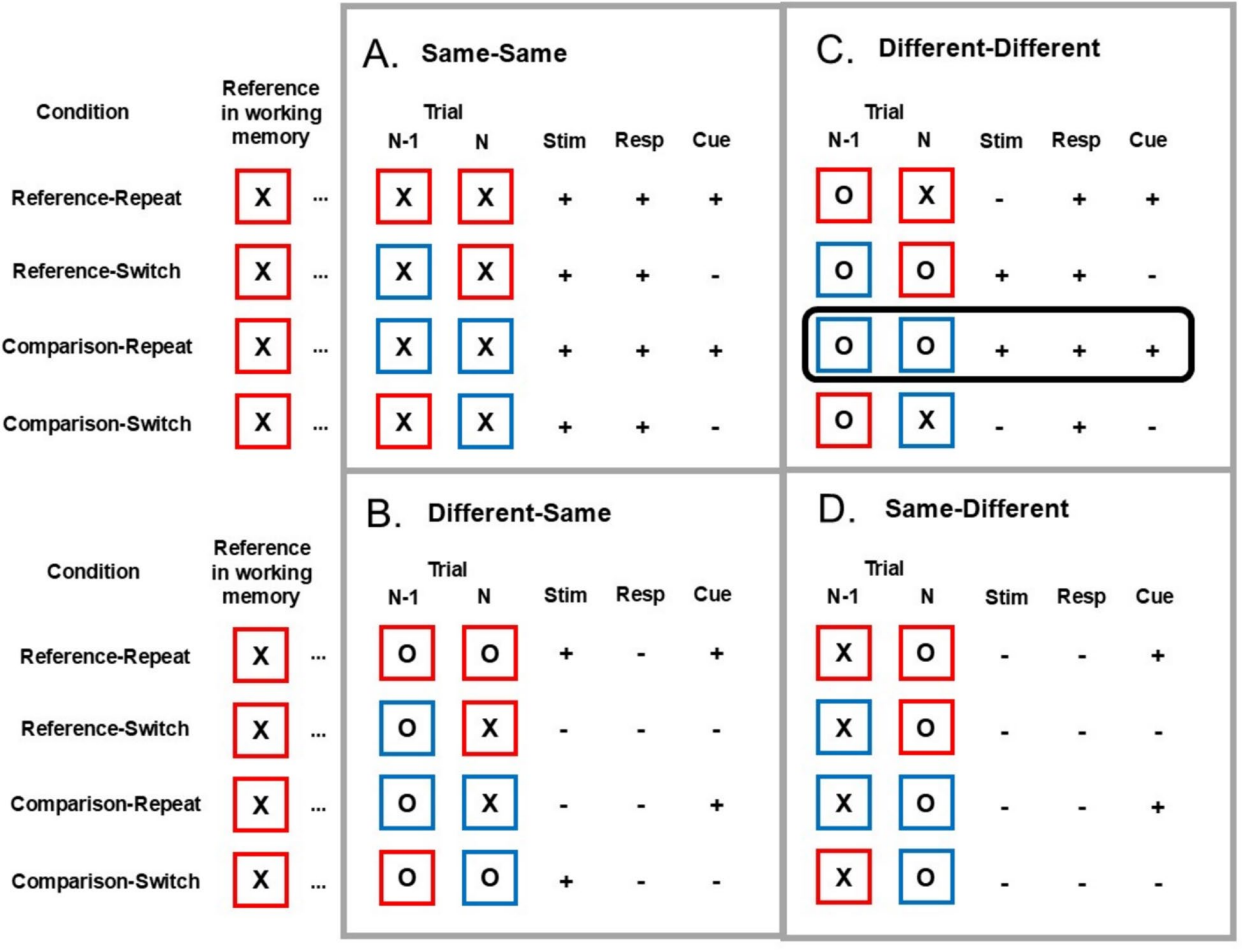
All combinations of stimulus (X/O), cue (red/blue frame), and response (same/different) alternations and repetitions with respect to the preceding trial. Repetitions are denoted by +; alternations are denoted by −. The **A–D** panels correspond to the response sequences: **A.** “Same” response preceded by “same.” **B.** “Same” response preceded by “different.” **C.** “Different” response preceded by “different.” **D.** “Different” response preceded by “same.” The reference held within working memory is X for all the illustrated trials. As demonstrated in **A.** and **C**., a full repetition of the stimulus, response and cue only occur in the comparison-repetition condition. See text for elaboration. Stim = stimulus; Resp = response. The critical condition that was sensitive to the ITI manipulation, different-different-comparison-repeat, is marked by a black frame. (Color figure online)

After establishing this effect, we turned to inspecting the data more closely in an exploratory manner, attempting to understand this pattern of results. We observed that the effects of intertrial interval seem to be moderated by the responses (whether “same” or “different”) that were carried out both in the present trial (trial N) and in the previous one (trial *N*−1). Specifically, the update-switch cost asymmetry in the short interval was most pronounced in one situation: trials in which the response “different” was indicated, following a “different” response in the previous trial. In these trials, performance was facilitated in comparison-repetition trials (i.e., a comparison trial preceded by a comparison trial), presumably since, in this condition, all three features—the cue, the stimulus, and the response—were repeated (see Fig. 3). Importantly, the response-time benefit in this specific condition was affected by the intertrial interval. While these three features were also repeated in two other cells of the design (same-same-reference-repeat, and same-same-comparison-repeat), these do not require removing the present stimulus from working memory (see General Discussion for details).

While this observation was admittedly post hoc, an ANOVA was conducted in which response-sequence (same-same, different-same, same-different, different-different; the first term described the response in trial *N*−1, and the second in trial *N*) was added to the previous factors (trial-type, intertrial interval, and update-switch). This four-level factor was used rather than entering the responses in trial *N* and in trial *N*−1 as two separate factors for the sake of simplicity (namely, to avoid a 5-factor design). The four-way interaction was significant, *F*(2.62,49.84) = 9.44, *MSe* = 8232.56,* p* < 0.001, η_p_^2^ = 0.33, BF = 774.58. Follow-up analyses probed the simple three-way interaction between trial-type, update-switch, and intertrial interval within each of the response sequences. This analysis confirmed the observation that the update-switch costs differed between the intertrial intervals mostly in the different-different response sequence, as explained above and reflected in the large BF (138.08; see Table 2).

**Table 2:** Experiment 1: Response-sequence analysis. *F*, *p*-values and BFs are presented for the simple three-way interaction between trial-type, update-switch, and intertrial interval, within each response-sequence

	Trial-Type*ITI*Update-Switch			Update-Switch: Short ITI			Update-Switch: Long ITI			
Response-Sequence	F(1,19)	p	BF	BF interaction	Comparison	Reference		BF interaction	Comparison	Reference
Same-Same	5.14	0.035	2.11	1.49	138 [86;191]	186 [110;262]		0.99	84 [29;170]	53 [7;100]
Different-Same	0.27	0.607	0.25	0.25	16 [− 20;51]	− 6 [− 79;68]		0.23	− 25 [− 63;12]	− 19 [− 77;40]
Same-Different	0.62	0.440	0.29	0.27	− 6 [− 81;70]	22 [− 44;88]		1.15	− 52 [− 101;2]	14 [− 44;72]
Different-Different	17.25	< 0.001	138.08	660.12	371 [273;470]	18 [− 77;114]		0.33	201 [139;265]	146 [51;242]

Update-switch costs (and 95% confidence intervals) are presented for comparison and reference trials separately for each response-sequence in each intertrial interval. BF_interaction_ refers to the Bayes factor of the simple-simple interaction between trial-type and update-switch, within each response sequence and intertrial interval. ITI = intertrial interval. The simple three-way interaction between trial-type, update-switch, and ITI is mainly observed in the short-ITI condition of the different-different response sequence.

#### Error rates

The mean overall error rate was 4.8% (95% CI [4.4; 5.2]). We implemented the three-way model on error rates as a dependent variable. No significant main effects or interactions were observed.

### Discussion

**Fig. 4:**
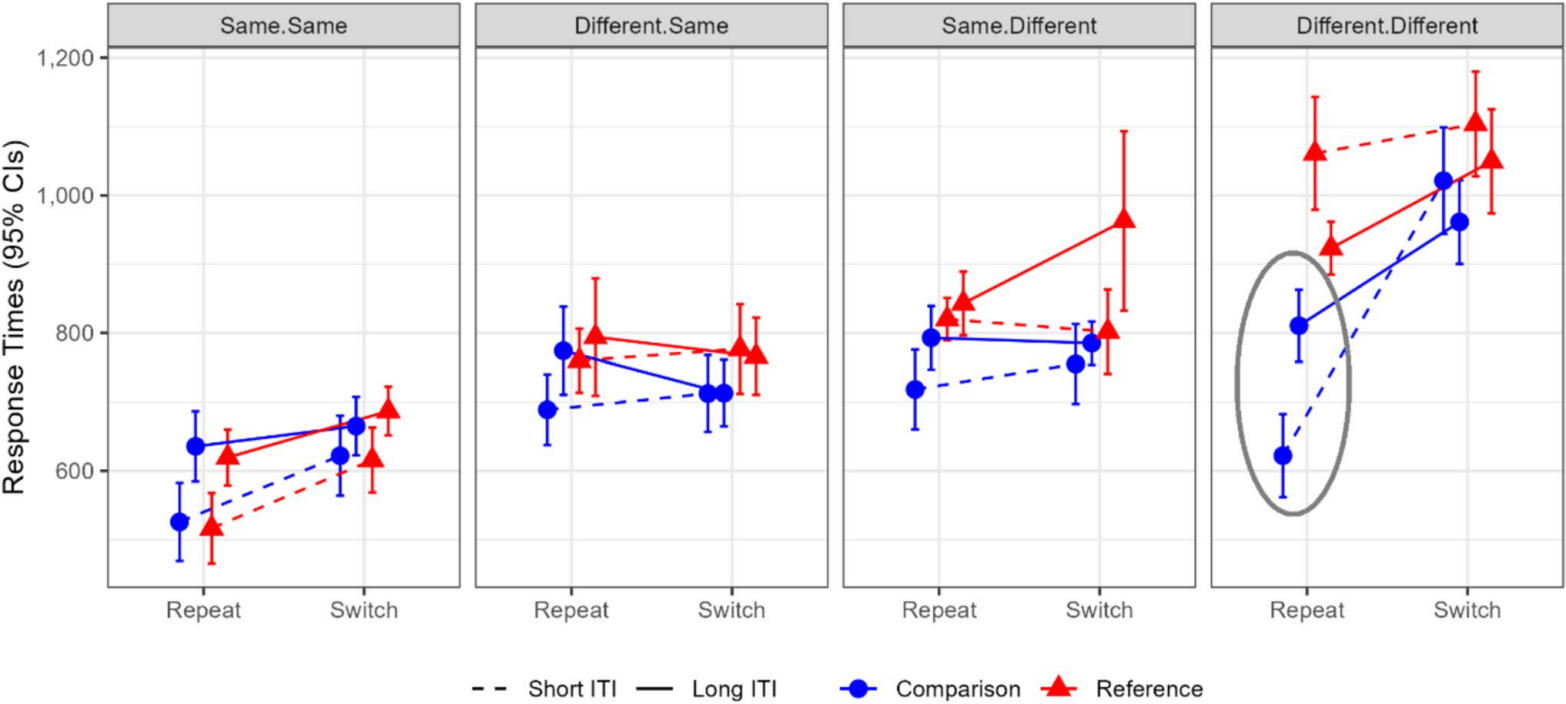
Experiment 1 results. Mean response times by update-switch, trial-type, intertrial interval, and response-sequence. Error bars denote 95% within-subject confidence intervals. The critical condition that was sensitive to the ITI manipulation, namely different-different-comparison-repeat (see Fig. 3), is circled in grey. (Color figure online)

In Experiment 1, we demonstrated in a within-subject design that “gate opening” costs—namely, switching from comparison to reference trials, are smaller than “gate closing” costs—namely, switching in the other direction. In other words, we show an underadditive interaction between trial-type and update-switch in short intertrial intervals but not in long intervals. Moreover, we exploratively analyzed the effect of response-sequence (i.e., whether participants repeat ‘same’/’different’ responses from one trial to the following one or switch between them) on this finding. We found that the underadditive interaction in the short intertrial interval was mostly driven by trials in which a ‘different’ response was repeated. In this condition, specifically on comparison-repetition trials, there is an response-time advantage caused by the repetition of cue, stimulus and response features (see Figs. 3–4).

Based on these findings, in the General Discussion we will propose a novel account of working memory updating in the reference-back task. However, before doing so, we present the results of a second experiment aimed at replicating these findings. Specifically, we conducted a second, larger-scale experiment in which intertrial interval was randomly manipulated on a trial-by-trial basis within a block, so that the upcoming ITI before the next trial could never be predicted. This manipulation ensured that the findings of Experiment 1 were not driven by a block-wise strategy.

## Experiment 2

### Method

#### Participants

Thirty-nine students (31 women; age range: 19–31 age, mean = 23.72, *sd* = 2.25) from Ben-Gurion University of the Negev participated in the experiment for course credit. Data collection for this experiment was completed in November 2021. No formal power analysis was conducted. All the participants were right-handed and reported having no neurological problems or learning disabilities. The study was approved by the Department of Psychology Ethics Committee, Ben-Gurion University of the Negev.

#### Procedure

The procedure in Experiment 2 was identical to the one used in Experiment 1, except for the intertrial interval, which varied randomly in each trial (1,000 vs. 4,000 ms, with equal probabilities).

### Results

#### Response times

**Fig. 5:**
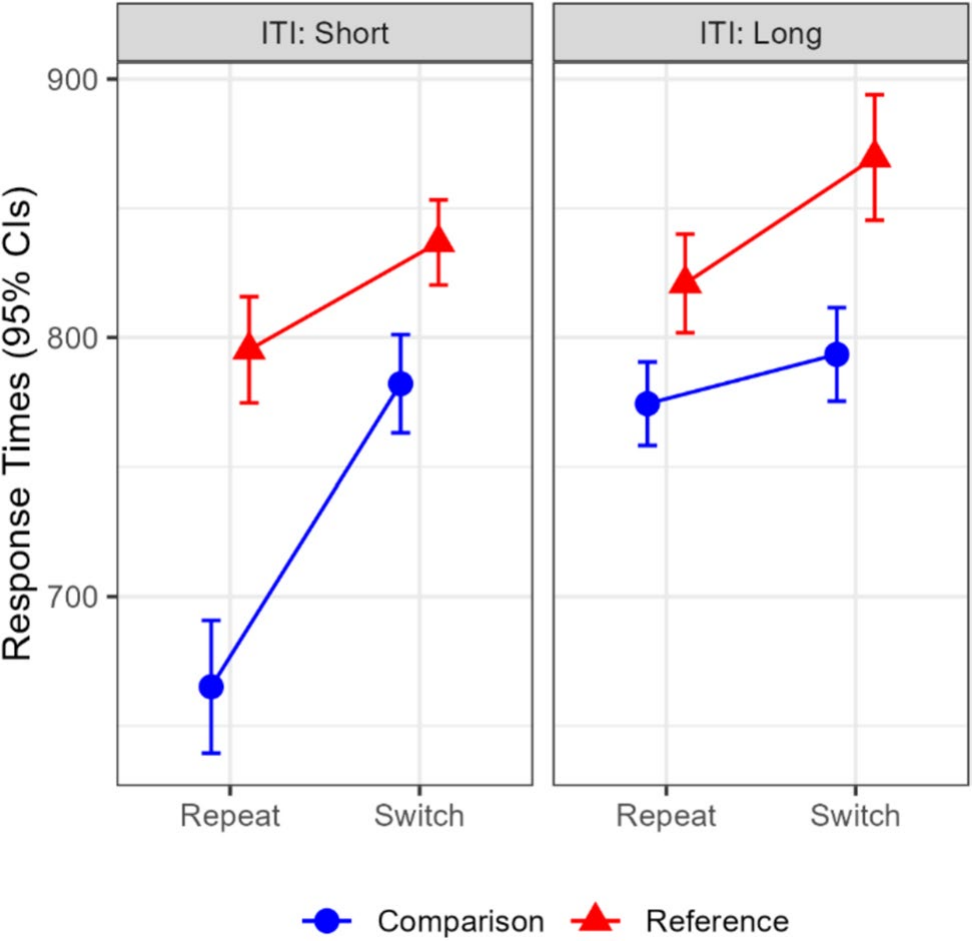
Experiment 2: Mean response times by update-switch and intertrial interval. CI = confidence interval; ITI = intertrial interval. Error bars denote 95% within-subject confidence intervals

As in Experiment 1, we started with a repeated-measures ANOVA with trial-type, update-switch, and intertrial interval. All main effects and interactions were significant. Importantly, the three-way interaction was significant, *F*(1,38) = 25.89, *MSe* = 2 083.28,* p* < 0.001, η_p_^2^ = 0.41, BF = 5310. The simple interaction between trial-type and update-switch was underadditive in the short intertrial interval, *F*(1,38) = 21.94, *p* < 0.001, η_p_^2^ = 0.37, BF = 1505.49, and—unlike in Experiment 1—slightly over-additive in the long interval, *F*(1,38) = 4.46, *p* = 0.041, η_p_^2^ = 0.01, BF = 1.29 (see Fig. 5).

**Fig. 6:**
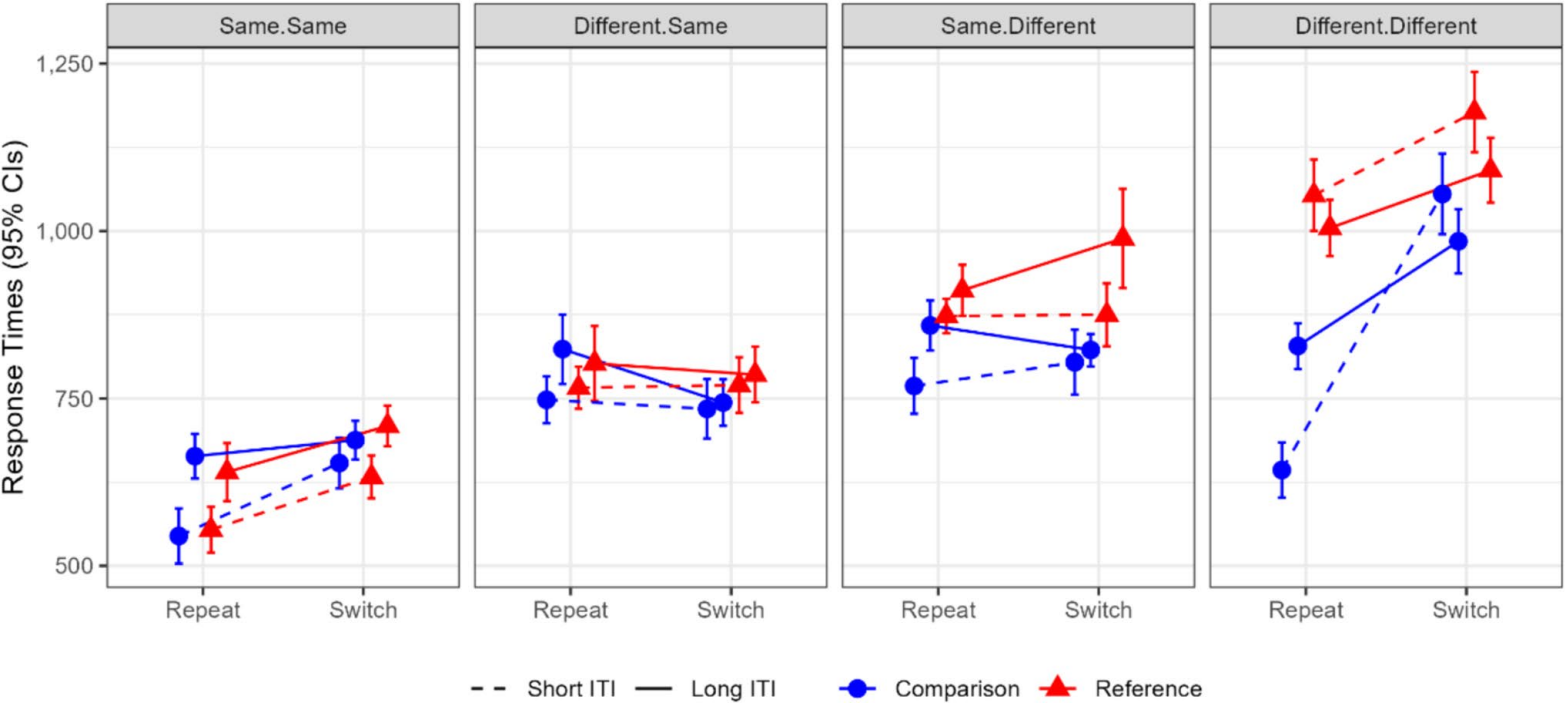
Experiment 2: Mean response times by update-switch, response-sequence, trial-type and intertrial interval. Error bars denote 95% within-subject confidence intervals

As in Experiment 1, we continued by conducting a second ANOVA in which the response sequence was entered as a fourth repeated-measure factor. One participant was removed from this analysis due to missing cells. Unlike Experiment 1, the four-way interaction did not reach significance, *F*(2.67,98.69) = 2.18, *MSe* = 14,570.38, *p* = 0.102, η_p_^2^ = 0.06, BF = 0.551 (see Fig. 6). However, our main finding regarding the simple three-way interaction in the different-different condition was replicated (see Fig. 6 and Table 3).

**Table 3:** Experiment 2: Response-sequence analysis

Response-Sequence	Trial-Type*ITI*Update-Switch			Update-Switch: Short ITI			Update-Switch: Long ITI		
	*F*(1,37)	*p*	BF	BF interaction	Comparison	Reference	BF interaction	Comparison	Reference
Same-Same	4.05	0.051	1.04	0.34	109 [74;144]	79 [50;107]	0.44	24 [− 3;51]	69 [17;120]
Different-Same	0.90	0.350	0.24	0.20	− 14 [− 56;29]	4 [− 40;48]	0.36	− 80 [− 142; − 17]	− 17 [− 74;41]
Same-Different	7.84	0.008	6.62	0.27	35 [− 15;86]	2 [− 42;46]	7.46	− 36 [− 75;2]	77 [− 1;155]
Different-Different	10.00	0.003	17.09	3982	412 [326;499]	124 [49;199]	1.08	156 [106;207]	86 [32;141]

*F*, *p*-values and BFs are presented for the simple three-way interaction between trial-type, update-switch, and intertrial interval, within each response-sequence.

Update-switch costs (and 95% confidence intervals) are presented for comparison and reference trials separately, for each response-sequence in each intertrial interval. BF_interaction_ refers to the Bayes factor of the simple–simple interaction between trial-type and update-switch, within each response sequence and intertrial interval. ITI = intertrial interval.

#### Error rates

The mean overall error rate was 4.8% (95% CI [4.5; 5.1]). In the three-way ANOVA model, the three-way interaction between trial-type, update-switch, and intertrial interval was non-significant, *F*(1,38) = 1.11, *MSe* = 4.51 × 10^–4^, *p* = 0.299, η_p_^2^ = 0.03, BF = 0.268, suggesting that speed–accuracy trade-off did not affect our response times analysis.

### Discussion

Experiment 2 used a larger sample size with a within-block intertrial interval manipulation, in which the participants could not anticipate the upcoming intertrial interval duration. The results replicated the key findings of Experiment 1, demonstrating an underadditive interaction between trial-type and update-switch in the short intertrial interval but an additive pattern in the long interval. In addition, we reconfirmed the observation of Experiment 1, that this effect was driven by the responses in the current and previous trials. Specifically, in trials where a ‘different’ response was preceded by a ‘different’ response, participants responded faster to comparison-repetition trials. In the General Discussion, we propose a novel account of the processes that take place in the reference-back task, which can explain this pattern of results.

## General discussion

We reported data from two experiments using the reference-back paradigm in which the intertrial interval was manipulated in a blocked (Experiment 1) and trial-by-trial (Experiment 2) design. Together, these experiments affirmed that switching to comparison trials is more costly than switching to reference trials when using a short intertrial interval (1,000 ms), but that these costs are equivalent when using a long intertrial interval (4,000 ms). Moreover, we found that the asymmetrical switch cost in the short intertrial interval was driven by trials in which a “different” response was repeated in a row. As we set out in more detail below, this pattern of observations demonstrates that the update-switch asymmetry in the short intertrial interval is not intrinsically related to the process of update-switching. We propose here that our findings can be best explained by assuming two different updating processes in working memory: an automatic, effortless one for context-free items and an intentional, effortful one for item-to-context bindings. In the following sections, we map out this proposal in detail and apply it to the results obtained in our two experiments.

Following Kessler et al. (2023), we suggest that the degree of attention that is required to enable response selection on an item (e.g., to make a same/different judgment) results in the obligatory encoding of this item into working memory. Accordingly, updating *item representations* in working memory is carried out as part of response selection and does not require any additional encoding process. Hence, it is automatic and relatively quick. By contrast, updating item-to-context bindings is slower and non-obligatory.

Based on the notion of updating as a by-product of attention, it follows that in each trial, regardless of the reference/comparison condition, the presented stimulus enters working memory. However, only stimuli that appear within red frames, namely in the “reference” condition, need to be maintained. Stimuli that appear in blue frames, on the other hand, need to be removed from working memory once their processing ends. This is because keeping them in working memory would interfere with the maintained reference stimulus. Removal of outdated or irrelevant information is a central process of working memory, which enables efficient use of its limited capacity by reducing interference from information that no longer needs to be maintained (see Lewis-Peacock et al., 2018, for a review). This removal process has been initially conceptualized as primarily operating on item-to-context binding (Lewis-Peacock et al., 2018), in line with embedded models of WM (Cowan, 1999). However, and crucial for our findings, Dames and Oberauer (2021) demonstrated that removal can be directed at single items as well.

The need for removal in the reference-back task arises only in comparison trials, where the participant does not need to remember the presented letter after making a response. Among these trials, the need for removal is most pronounced when the letter is different from the reference held in working memory, namely when the (correct) response is “different.” Removal is a time-consuming process (Oberauer, 2001), which may not be completed during the intertrial interval before the next trial appears. Accordingly, when the new trial appears before the removal process is complete, the processing of the stimulus in that trial is affected by the unremoved information that is still in working memory. This is the reason for the facilitation in short intertrial intervals that we see in comparison-repetition trials, where the response is “different,” following another “different” response. As demonstrated above, this specific condition drives the interaction between trial-type and update-switch and its sensitivity to the intertrial interval.

**Fig. 7:**
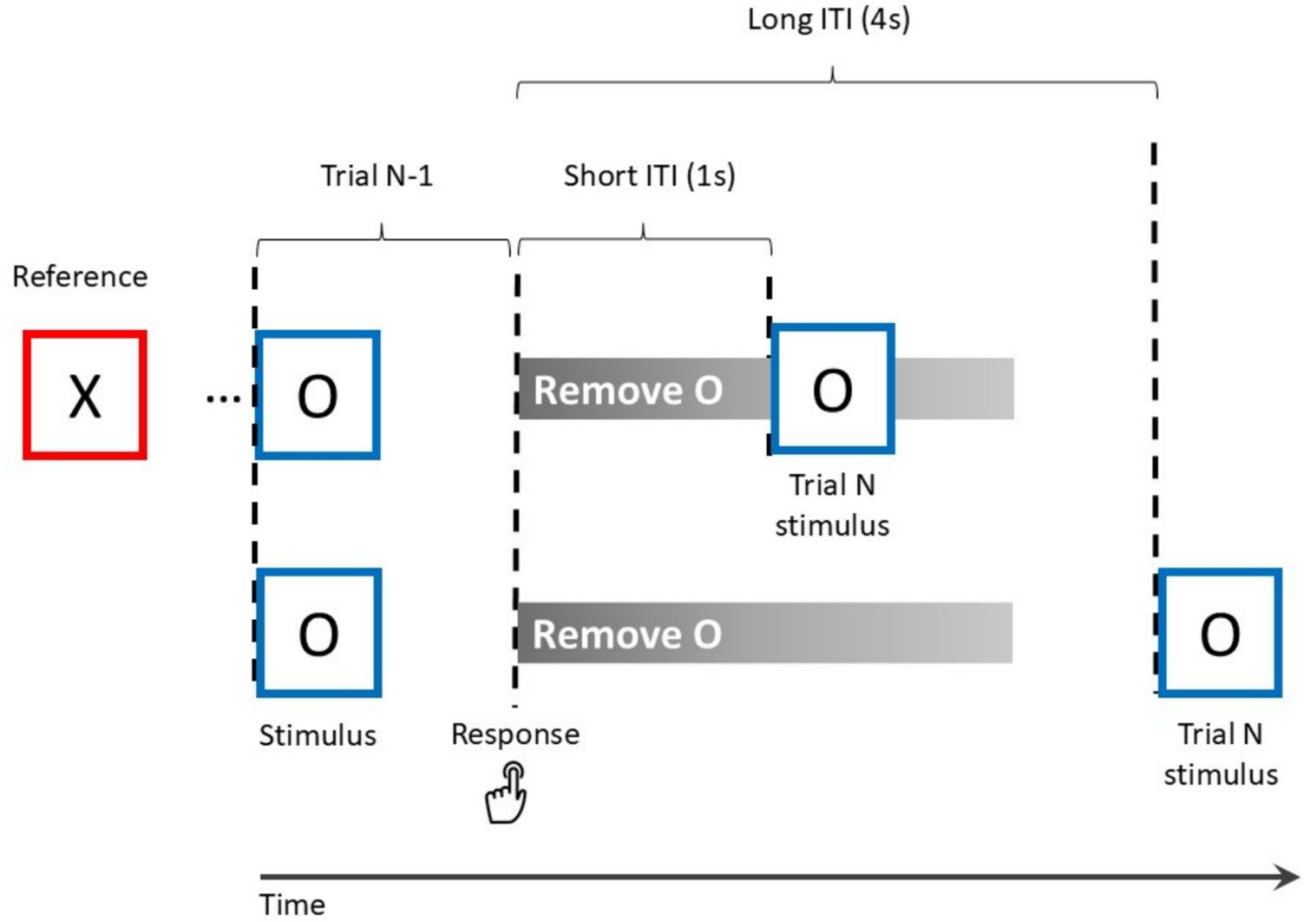
Contents and processes in working memory in the different–different response sequence. Assume that X is the reference maintained in working memory, and the stimulus O appears in Trial *N*−1 within a blue frame. O is encoded into working memory as part of the response selection process of Trial *N*−1. Once the response “different” is indicated, it should be removed from working memory. Since removal takes around a second, in the short intertrial condition, the following trial (Trial *N*) can appear before the removal is complete so that O is still in working memory. This leads to the facilitation of processing the probe O in Trial N. This facilitation does not take place in long intertrial intervals, in which removal is completed before the onset of Trial *N*. (Color figure online)

For a concrete example, assume the reference letter in working memory is X, and the probe O appears on the screen (see Fig. 7). In this case, X is bound to the “context” or “role” of being the reference item. The participant needs to respond “different”, since the probe is different than the reference. As part of this same/different decision, attention is directed to the probe, leading to its encoding into working memory. At this point, the probe O is temporarily maintained in working memory as an unbound item. If this is a reference trial (i.e., the frame is red), the binding between X and the “reference” context should be now removed, and a new binding between O and “reference” is then created. These two processes are responsible for the slowing observed in reference trials compared with comparison trials, namely to the updating cost. However, if this is a comparison trial (the frame is blue), then the unbound item O must be removed once the response (“different”, in our case) is selected.

The duration of removing information from working memory is estimated to be up to one second (Ecker et al., 2010; Oberauer, 2001). Until this process is completed, the to-be-removed item still affects performance. Continuing the above example, consider a situation where the following probe (Trial *N*) is again O, and it is presented within a blue frame (Fig. 6). This is the critical *comparison-repetition-different-different* condition described above. Note that in this condition, all the features of trial N, namely the cue, the probe and the response, are repeated. The benefit of this repetition, however, is mostly pronounced when the probe of trial *N*−1 is still in working memory, namely, its removal was not complete yet. Given that removal takes around 1 s, on average, this situation takes place when trial N appears after a short intertrial interval, but not after a long one. Notably, the effect of facilitated updating for repeated or related items in short intertrial intervals, and the reduction of this benefit in long intervals, was also documented in previous studies (Ecker et al., 2014a, 2014b; Ecker et al., 2014a, 2014b; Lendínez et al., 2011).

### Broader implications

The finding that updating is faster than not updating when the memory set includes one item only (Kessler et al., 2023) seems to be at odds with the gating model and literature on executive functions literature, which regard updating as a controlled and effortful process. In addition, our present proposal that attending to the probe leads to its encoding into working memory in both the reference and comparison conditions calls for a revised interpretation of this task for two reasons. First, it is at odds with our previous view that updating only takes place in reference trials. Second, it seems to undermine the idea that alternating between the conditions requires the opening/closing of the gate to working memory.

These discrepancies can be reconciled by making a clear distinction between updating a single item and an item-to-context binding. As a side note, we will say that it is tempting to refer to these as encoding versus updating, respectively. However, using these terms risks being incompatible with previous work, including ours, and hence warrants more consideration. Based on this distinction, updating in the reference-back task refers to item-to-context binding because reference trials require modifying the item that is bound to the “role” of being the reference. In our view, the role of gating is not to determine whether an item enters working memory but rather to control the binding of this item to a context representation in working memory. In other words, gating is implicated between two “levels” of working memory, context-free items and bound item-to-context associations. The opening and closing of the gate, as operationalized by alternating between the reference and comparison conditions, coordinates the binding of attended items to the “role” of being the reference. Keeping the gate closed in comparison trials prevents the attended (and, as a result, “encoded” into working memory) probe from updating the maintained reference.

In sum, our findings and interpretation integrate two views on working memory updating, i.e., the view that updating requires effortful gating, on the one hand, and that it is an automatic by-product of attention, on the other hand. More precisely, the distinction between context-free and context-bound items that we have elaborated on here implies automatic updating for all items—indeed as a byproducts of attention—while still requiring the presence of a working memory gate to protect context-bound items from being overridden by context-free items, which is an effortful process. This synthesis resolves previous inconsistencies in the literature that stem from not explicitly making this distinction.

## Data Availability

All data and analysis code can be found on our Open Science Framework profiles (https://osf.io/7zspj/).

## Footnotes

1 The exact role of the prefrontal cortex in working memory maintenance is still debated. Whereas early work suggested that working memory representations are actively held in the prefrontal cortex, newer work suggests that the prefrontal cortex recruits sensory representations by directing attention toward them (e.g., `Postle, 2016). This debate is beyond the scope of the present paper.
